# Addressing the health human resources crisis: Strategies for retaining women health care professionals in organizations

**DOI:** 10.1371/journal.pone.0293107

**Published:** 2024-06-13

**Authors:** Abi Sriharan, Nigar Sekercioglu, Whitney Berta, Sylvain Boet, Audrey Laporte, Gillian Strudwick, Senthujan Senkaiahliyan, Savithiri Ratnapalan

**Affiliations:** 1 Krembil Centre for Health Management and Leadership, Schulich School of Business, York University, Toronto, Canada; 2 Institute of Health Policy, Management and Evaluation, Dalla Lana School of Public Health, University of Toronto, Toronto, Canada; 3 Department of Anesthesiology and Pain Medicine, The Ottawa Hospital, University of Ottawa, Ottawa, ON, Canada; 4 Department of Innovation in Medical Education, University of Ottawa, Ottawa, ON, Canada; 5 Ottawa Hospital Research Institute, Clinical Epidemiology Program, Ottawa, ON, Canada; 6 Institut du Savoir Montfort, Ottawa, ON, Canada; 7 Centre for Addiction and Mental Health, Toronto, Canada; 8 Hospital for Sick Children, Toronto, Canada; Bangladesh Open University, BANGLADESH

## Abstract

Globally, healthcare systems are contending with a pronounced health human resource crisis marked by elevated rates of burnout, heightened job transitions, and an escalating demand for the limited supply of the existing health workforce. This crisis detrimentally affects the quality of patient care, contributing to long wait times, decreased patient satisfaction, and a heightened frequency of patient safety incidents and medical errors. In response to the heightened demand, healthcare organizations are proactively exploring solutions to retain their workforce. With women comprising over 70% of health human resources, this study seeks to gain insight into the unique experiences of women health professionals on the frontlines of healthcare and develop a conceptual framework aimed at facilitating organizations in effectively supporting the retention and advancement of women in healthcare frontline roles. We used grounded theory in this qualitative study. From January 2023 to May 2023, we conducted individual semi-structured interviews with 27 frontline HCWs working in Canada and representing diverse backgrounds. The data underwent thematic analysis, which involved identifying and comprehending recurring patterns across the information to elucidate emerging themes. Our analysis found that organizational, professional, and personal factors shape women’s intentions to leave the frontline workforce. Reevaluating organizational strategies related to workforce, fostering a positive work culture, and building the capacity of management to create supportive work environment can collectively transform the work environment. By creating conditions that enable women to perform effectively and find satisfaction in their professional roles, organizations can enhance their ability to retain valuable talent.

## Introduction

Worldwide, health systems are currently grappling with a pronounced crisis in health human resources, characterized by elevated rates of premature retirements and escalating levels of burnout [1–4]. This situation is particularly poignant for women, who currently comprise 70–80% of the global healthcare workforce [5]. The impact of COVID-19 has further exacerbated these challenges [6, 7]. The consequences of this crisis are far-reaching, including long patient wait times, low patient satisfaction, and increasing patient safety incidents and medical errors [8, 9]. The retention of healthcare professionals has become a major concern for healthcare organizations, as the demand for healthcare services continues to increase [1]. Given the global nature of the health human resources crisis, there is heightened competition in the market for the already limited pool of healthcare professionals. As a result, health care organizations are currently engaged in significant recruitment efforts to meet their human resources needs [10–13]. However, recruitment efforts alone are not sustainable. It is equally important to retain the current workforce, as well as the newly recruited clinical staff [14].

The existing literature on the retention of health workforce is notably constrained. Primarily, it lacks a gendered approach to analysis, often providing insights predominantly from the standpoint of attracting nurses or physicians to rural areas. Factors, such as the work environment, recognition, higher salary, mentorship, and professional development are recurrently identified as pivotal components for fostering job satisfaction and retention in these contexts [15–18].

Work-related experiences of women in health care are unique [6]. Gender bias, violence and incivility remain persistent problems in health care [19, 20]. This is further exacerbated by the demanding nature of healthcare work, which involves long hours, including after-hours and weekend work; emotional labor; complex patient demands, and a relentless pace [6, 21–23].

## Research question

What influences the turnover intentions of women working in frontline healthcare roles? How can organizations effectively retain women in frontline positions?

## Methods

### Design

We undertook an exploratory grounded theory study [24, 25] utilizing cross-sectional interviews to understand how women in healthcare experience work and explore what employers can do to support them at work. The study was guided by the best practices outlined in the SRQR guidelines [26].

### Research team

The research team comprised health professionals, health services researchers, and organizational researchers. This qualitative research study was undertaken as a component of a broader research initiative aimed at comprehending women’s experiences in healthcare. All interviews were consistently conducted by the same interviewer (PhD level), who underwent training based on the interview guide. Participants in the study were drawn from various organizations in Ontario, and there were no direct relationships between the participants and the research team.

### Setting

Canada’s health care system is primarily funded by the government, with around 70% of its financing coming from public sources. This publicly funded system ensures that all residents have access to essential medical services. Most physicians work as independent contractors in health care facilities and receive payments through a single-payer system. A wide range of health care professionals, including nurses and allied health professionals, are employed by hospitals, clinics, long-term care facilities, and home health care agencies. More than 39% of nurses in Canada have expressed intentions to leave their positions within the next one to five years, with nearly 45% of nurses under the age of 30 indicating their intention to depart within one year [27]. The study took place in the province of Ontario in Canada.

### Sampling technique and recruitment of participants

We employed a purposive sampling methodology to address our research objectives. We established specific criteria for participant inclusion in our recruitment process: (a) self-identifies as a woman; (b) is a health care professional in Ontario, which includes licensed physicians, registered nurses, registered practical nurses, nurse practitioners, and other health care providers such as personal support workers and physician assistants; and (c) has engaged in direct patient care within a health care delivery setting, either currently or within the past 24 months. Our recruitment strategy encompassed email campaigns, social media posts, listservs, and recruitment flyers through various channels within Ontario-based hospital networks. Interested participants were invited to complete a screening form to ensure they meet the eligibility criteria. We continued our recruitment efforts until we reached a point of data saturation [28]—that is, where no new substantial insights were emerging.

### Data collection

All interviews were conducted between January 2023 and May 2023. We used a semi-structured open interview guide to allow participants to reflect on their healthcare professional experiences. The primary interview inquiries concerned participants’ experiences, expectations of their professional roles, and what organizations can do to support them.

Before the interviews, we emailed the eligible participants an electronic informed consent form and clarified that participants had the right to withdraw from the interview at any point. Once we received the electronic consent form, interviews were scheduled at a time convenient to the participant. All interviews were conducted via Zoom to maintain consistency, with one interviewer trained in the interview guide conducting all interviews. All interviews were conducted in English. Interview recordings were stored in a protected electronic database in accordance with the ethics requirements. All participants were invited to complete a separate optional structured personal data form, which gathered information about demographics (age and cultural background) and lifestyle details (current employment status, annual income, health care work duration, and parenting status). Participants received a copy of the draft manuscript to review and verify the findings.

### Ethics

The study commenced after ethics approval from the University of Toronto’s Research Ethics Board (REB 43932). Participants who completed the research interview were given $60 gift cards as a token of appreciation for completing the interview.

### Data analysis

Interviews were recorded, professionally transcribed verbatim using MAXQDA transcription tool, deidentified, manually cleaned and reviewed for accuracy. Transcripts were not shared with the participants. Using grounded theory, we conducted open coding on emerging data from the interviews. In the open coding process, we systematically divided the data into smaller segments and assigned descriptive codes to them. This approach allowed us to explore various ideas and concepts within the data without any preconceived notions.

Once all interview transcripts were coded, reports were generated that collected all the quotations assigned to the same code. We then used a thematic analysis approach to identify and analyze repeated patterns across data and explain emerging themes [29]. First, through an iterative process, the code reports were examined together to develop axial codes. We used axial coding to establish connections and relationships between the initial codes, and we organized them into broader themes based on our guiding research questions. We then analyzed how the themes are related to each other to identify the core concepts that underlie the data. We kept detailed notes of the decisions, questions, and comments.

To increase the rigor of our analysis, two independent researchers (NS, SS) coded all the data. A third researcher (AS) reviewed all the codes, themes, concepts, and quotes, and then three researchers (AS, NS, SS) met to resolve any discrepancies.

## Results

We included 27 participants in this study (nine physicians [33.3%], 15 nurses [55.5%], and three [11.2%] allied health care professionals). We received 25 responses to the demographic survey. Participants represented diverse age, racial, experience, and income backgrounds (Table 1).

**Table 1:** Participants characteristics.

**Eligibility Criteria**	
In the past 24 months worked in a regulated health profession in Ontario	
Self-identify as a woman	
Within the past 24 months had worked in the frontline clinical care	
**Total Number of Participants**	27
**Total Number of Participants who completed the demographical questionnaire**	25
**Employment Status**	
Full-time	15
Part-time	7
Not presently working/Retired	2
Not disclosed	1
**Years of Experience**	
1 to 5 years	8
6–10 years	12
11–15 years	4
More than 15 years	1
**Annual Income**	
Less than $50,000	1
CAD $50,000- $ 100,000	7
CAD $100,000–150,000	5
CAD $150,000–200,000	7
CAD $200,00+	2
Prefer not to say	2
**Cultural Background**	
First Nations or Indigenous	4
Asian	5
African	11
European	2
Prefer Note to Say	3
**Number of Dependents**	
None	8
1–2	14
3–4	3
**Age**	
Less than 30	3
31–40	12
41–50	10

### What influences the turnover intentions of women in frontline healthcare roles?

We grouped the participants responses into three major themes: organization-related factors, profession-related factors, and personal-related factors.

#### Organizational factors

Workload emerged as a predominant concern among participants, a sentiment that persisted beyond the challenges posed by the COVID-19 pandemic into the post-pandemic period. Most highlighted that existing staff members are grappling with heightened workload pressures due to significant staffing shortages. Moreover, the increased workload necessitated extended work hours, resulting in limited personal time and contributing to emotional exhaustion. There was a pervasive sense that their compensation does not adequately reflect their workload demands. Participants perceived biases from their leaders, with preferences often favoring individuals based on gender or race in terms of opportunities for advancement and access to career advancements. Participants noted a need for more understanding from leaders regarding the distinctive experiences of women, when imposing unrealistic expectations, such as unilaterally determining work schedules without prior consultations. Moreover, several participants highlighted that their work environments do not facilitate open discussions about their unique challenges. Many expressed fear that openly addressing these issues could lead to negative consequences. Table 2 provides a summary of sub themes and illustrative quotations.

**Table 2:** Organization-Related factors.

**Workload**	*It’s been a quite a burden from staffing perspective in order to keep up with our the demands that are happening*.*(P25)*
**Fair Compensation**	*people are taking more than they can do they have to carry on particular work that was not your work*, *and not paid for*. *so the work is being increased*. *they quit*. *(P7)*
**Work Conditions**	*I found it hard working the evening shift*, *sometimes because I wouldn’t see my family*. *And not having that option to ask to not be scheduled in that shifts are often not understood by the management (P21)*
**Bias**	*Yeah*, *most people sometimes think they’re not being represented appropriately*, *and they’re not being recognized most times which force women to leave workforce at higher rates from men (P8)*
**Work environment**	*it is more how your work environment manager or work colleagues and how you are treated in the workplace can contribute to the resignation of some women(P11)*

#### Profession-related factors

The participants in the study revealed that the COVID-19 pandemic had made them become more aware of the demands of their profession, the professional hierarchies within healthcare, and the limited autonomy of their professional roles. They mentioned that while administrative colleagues and clinical leaders could work from home, they had to stay at the healthcare facilities due to patient care requirements. This situation became more complex as they had to accommodate their executive colleagues working remotely. Moreover, the restrictions on how care could be given and the type of resources available made it tough for them to negotiate between the best care based on their clinical judgment, what patients expected from them, and what the organizational and systems structures allowed them to do. Despite all the difficulties, participants highlighted their passion and sense of purpose for clinical work, which motivated them to continue in the face of adversity. Table 3 provides a summary of themes and illustrative quotations.

**Table 3:** Profession-Related factors.

**Expectations**	*“I think the culture*, *the culture in medicine is to just keep doing*.*” (P20)*
**Hierarchy**	*“trying to assert my authority and expertise*, *and having people see me look as if they think I’m too young or you know I they don’t believe me or trust me”(P24)*
**Autonomy**	*We are expected to work a lot more nights without any consent like this*. *You’re on the schedule*, *that’s it (P24)*
**Commitment to Clinical Work**	*I think it my passion*, *the passion I have for my field*. *That was what helped me*. *(P14)*

#### Personal factors

The participants opened up about the difficulties they faced in achieving a work-life balance while fulfilling their familial responsibilities. They pointed out that women face additional challenges both at home and in the workplace, compared to male colleagues, and any unfairness they experience at work can have detrimental effects on their personal lives, potentially leading them to consider quitting their jobs. Furthermore, the pandemic has amplified concerns about safety among the participants. Witnessing their colleagues and patients contracting COVID-19 and suffering life-altering consequences or even death has caused them to feel stressed, anxious, and traumatized. Additionally, the participants shared increase in recruitment efforts for better paying opportunities and the departure of their coworkers for better opportunities elsewhere, has tempted them to consider exploring alternative job prospects. Table 4 provides a summary of themes and illustrative quotations.

**Table 4:** Personal-Related factors.

**Safety Factors**	*for frontline workers*, *we can’t work from home*, *there’s no quarantine for us always at the hospital being exposed to patients we are stressed especially for me*. *I live in a household with a lot of people*, *and my grandparents were there at that time*. *(P27)*
**Values**	*Making sure that you are able to balance*, *your work*, *and also taking care of the house(P1)*
**Gendered Experience**	*There are multiple hats not saying men don’t have that but I also think there’s a lot of things women go through the Men don’t*. *But I find that our coping mechanisms*, *the way we take burdens on the way we perceive chaos in the way is different*. *We are just biologically different*. *And we need to recognize that (P26)*
**Economic Reasons**	*I think some people who left the workforce were able to*, *because they had support*. *They weren’t the sole income to their family like that’s it’s a privileged spot to be able to leave the workforce which is not open to everybody (P20)*
**Fairness**	*you know*, *you work a lot you expect much in return*.*(P12)*

### How can organizations effectively retain women in frontline positions?

Organizations can effectively retain women in frontline clinical care by addressing their \ human capital strategy, organizational culture, and leadership.

#### Human capital strategy

Participants emphasized the significance of fair and equal compensation and benefits, such as paid leave and access to free medical services, as pivotal factors that motivate them to stay in the workforce. There was a perception that women are not paid equally for their work efforts. To retain them in the workforce, organizations should ensure fair compensation, respectful work conditions, equitable benefits, and policies that are inclusive of women’s unique circumstances. In addition to the compensation and benefits, participants identified the importance of organizational investment in their personal and professional growth through professional development training that could substantially enhance their skill sets and their opportunities for advancement. Participants emphasized the significance of virtual work. They highlighted the potential of using remote patient monitoring and remote work for administrative tasks as strategies to offer a more flexible work environment for women working in frontline clinical roles. Table 5 provides a summary of themes and illustrative quotations.

**Table 5:** Strategic investments related to human capital.

**Compensation**	*I think people should just be paid according to the efforts (P 16)*
**Inclusive**	*supporting families and creating friendly policies that would not be gender biased or race biased and help ensure that employees do not have to choose between career and family*. *(P8)*
**Workload**	*reduce the workload it will encourage people to continue to work (P3)*
**Virtual Work**	*I think telehealth was kind of more providing a better environment for a health care professional*, *and especially nurses and women*. *(P8)*

#### Organizational culture

Participants expressed the critical importance of creating a workplace culture that recognizes individuals’ contributions in team-based care. They highlighted the significance of being treated respectfully and having their capabilities trusted by their team members. Moreover, they stressed the need for a safe space where they can openly communicate their concerns without any fear of discrimination based on their role hierarchy, gender or race. The participants also emphasized that offering opportunities to engage in key decision-making regarding patient care or organizational initiatives is essential for making them feel respected and valued by the organization.

Table 6 provides a summary of themes and illustrative quotations.

**Table 6:** Strategic investment related to organizational culture.

**Peer Support**	*“having a supportive environment is so important to me*. *Where I won’t be affected by one patient*, *and I can go home without feeling stressed” (P27)*
**Engagement**	*I feel like our voices should be heard*, *because sometimes we found ourselves in the settings where voice of us not heard*. *People sometimes think that the majority of women are doing ok but while they are facing a lot of issues (P2)*
**Respect**	*there is gender or racial bias*. *Organizations need to be culturally equal and acknowledge that all people work*. *People need to feel they are not alone and that they are being appreciated*. *(P 6)*

#### Leadership

Participants highlighted the pivotal role organizational leaders can play in showcasing their commitment to fostering a thriving work environment for women. This involves demonstrating awareness of the unique challenges faced by women managing both work and personal responsibilities. To achieve this, leaders can create a safe and open environment where women feel comfortable expressing their needs and concerns. Moreover, leaders can significantly contribute by providing mentorship and opportunities for professional advancement, fostering transparent communication about decisions and growth prospects, and cultivating positive work relationships that actively support and empower women. Table 7 provides a summary of themes and illustrative quotations.

**Table 7:** Strategic investment related to leadership.

**Feedback**	*constructive feedback are necessarily*. *I remember feedback as always when they try to criticize someone or something*. *(P27)*
**Empathy**	*for me*! *I know that the work environment may not accept my own life they are not fair to women*. *but if you ask me that the general manger should consider the workload for women*, *they should reduce the workload for women*. *(P11)*
**Advancement Opportunity**	*It’s because the representation of women in healthcare leadership limited specially for women of color*.*(P5)*

## Discussion

We conducted an exploratory grounded theory study to gain insights into women’s experiences in healthcare and the strategies to retain them. The grounded theory approach facilitated the organic emergence of themes from the participants’ perspectives. Our research makes three critical observations related to the turnover intentions of women in healthcare and what organizations can do to support them.

First, our study shows that the reasons for women leaving frontline health care are multifaceted and influenced by three interconnected factors- personal, profession-related, and organizational. While personal and profession-related factors may not be directly controlled by organizations, there is an opportunity for organizations to invest in management practices to support women in the workplace. This includes implementing human capital strategies like equitable compensation for women, supporting professional advancement, and transforming organizational culture to establish a safe environment for women to express their needs.

This finding is consistent with the turnover theory, which postulates employees who receive substantial support from their organization experience elevated levels of job satisfaction. They often maintain a balance between work and personal life. This in turn results in their unwavering commitment to their organizations, and are distinctly less predisposed to entertain thoughts of relinquishing their current employment positions [30].

Secondly, our study identified a significant theme concerning the perception of fairness related to human resource practices such as organizational rewards, incentives and advancement opportunities. This observation resonates with the principles of social exchange theory, emphasizing that employees who experience equitable treatment, support, and recognition from their organizations are more inclined to feel a sense of obligation and loyalty [31].

Thirdly, the study underscores the critical role of leaders in shaping a positive employee experience through their ability to demonstrate empathy, provide constructive feedback, and facilitate professional growth opportunities for team members. It is crucial to recognize that while the human capital strategy involves developing policies and procedures for fair compensation, inclusivity, virtual work opportunities, and effective workload management, leaders at both the organizational and team levels are essential in implementing these strategies. Moreover, fostering an organizational culture characterized by peer support, engagement, and respect for all individuals is also within the realm of leadership responsibility. This finding aligns with emerging research on leadership theory, emphasizing leaders who inspire and motivate followers by articulating a compelling vision, fostering trust through open communication, and committing to shared values. Such leaders drive organizational success and enhance the well-being and growth of individuals within the team [32].

**Fig 1:**
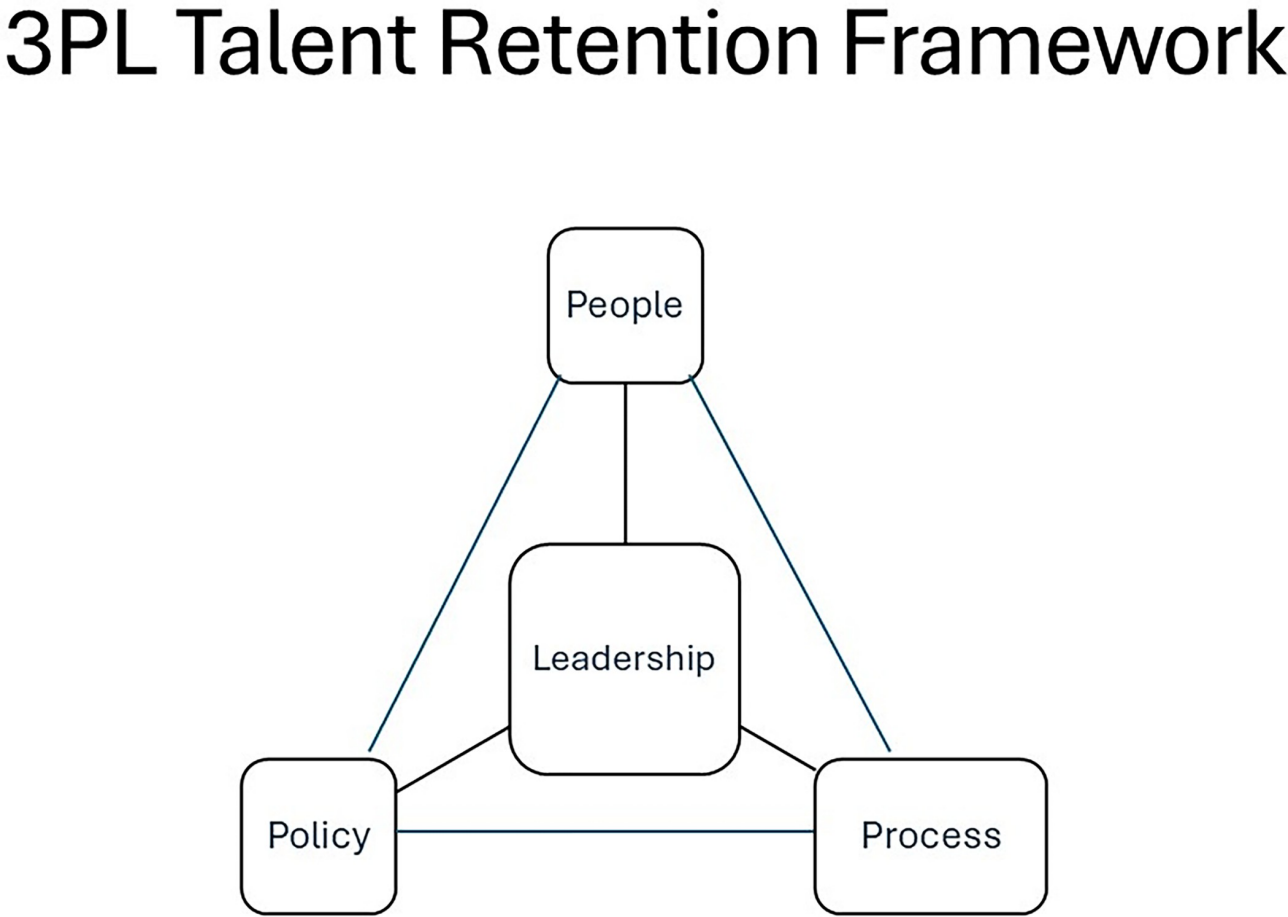
3PL talent retention framework.

Building on these findings, we present a conceptual framework aimed at assisting organizations in evaluating and aligning their endeavors to retain women healthcare professionals. Illustrated in Fig 1, this framework encompasses three interlinked domains: Policies, Practice, and People.

### Policies

Our study highlighted human resources policies within an organization must prioritize equitable compensation and reward distribution, ensuring the absence of gender pay disparities. Additionally, fostering an inclusive environment conducive to professional growth is essential.

### Practice

A conducive environment that fosters peer support, respect, and inclusive decision-making processes enhances employees’ commitment to the organization.

### People

The interplay among individuals, particularly the dynamics between employees and team leaders and among peers, significantly influences employees’ perception of a supportive work environment.

At the core of these components are leadership behaviors that prioritize employee development. This entails leaders who actively offer constructive feedback to support individuals in maximizing their performance, guide them in identifying growth opportunities, acknowledge and respect everyone’s unique experiences with empathy, and cultivate a safe environment conducive to open engagement.

Healthcare organizations are currently facing significant workforce challenges, particularly in recruiting and retaining clinical professionals. Traditionally, within health care organizations, talent management tasks such as compensation, benefits, and training fall under the purview of specialized human resource professionals. Complementing talent recruitment efforts led by human resources, a dedicated focus on enhancing managerial strategies to retain existing healthcare staff is crucial for strategic human resources planning in healthcare. Implementing the 3Ps Talent Retention Framework offers a structured approach for organizations to assess their current workforce strategies and cultivate a supportive work environment tailored to the needs of women health professionals.

However, it is essential to understand the limitations of this framework. Firstly, the framework is based on qualitative research that was conducted solely on women health professionals working in Ontario, Canada. This region has a predominantly publicly funded healthcare system and universal healthcare coverage. Additionally, the study was carried out in early 2023, after the resumption of regular healthcare operations post-COVID. Therefore, we acknowledge that the responses of the participants may have been influenced by the lingering effects of the pandemic.

As a result, the application of the framework in a specific organizational context should be contextualized to the local organizational or health systems situation.

Future research on retention of women in healthcare should continue to refine 3PL framework by applying the framework in different organizational or health systems context and quantitatively exploring the moderating relationships between the identified factors.

## Conclusion

Navigating frontline clinician role poses myriad challenges for women, placing the onus on organizations to cultivate supportive work environments for women to stay in those roles. In conclusion, it is evident that organizations can effectively nurture and retain their valuable human resource talent through a strategic reevaluation of organizational policies and processes. By prioritizing investments in people leadership, they can cultivate a supportive work environment conducive to addressing retention challenges.

## Supporting information

S1 File (DOCX)
