# Supplementary material for: Addressing the health human resources crisis: Strategies for retaining women health care professionals in organizations
Source: PLoS One. 2024 Jun 13;19(6):e0293107. doi: 10.1371/journal.pone.0293107 (PMC11175397; doi:10.1371/journal.pone.0293107)
Supplement: S1 File — (DOCX) [file pone.0293107.s001.docx]

**SUPPLEMENTARY MATERIAL**

Why they Persist in Health Workforce: A Mixed Method Study to Characterize and Examine the Experiences of Women

Interview Guide

BACKGROUND

First, let us THANK YOU for agreeing to participate in our research project. We are (NAMES) and we are researchers from the Institute of Health Policy, Management and Evaluation. We have reached out to you as part of a project we are doing to study how women in healthcare navigate work and work related burnout.

As part of this study, we are interested in collecting information from women who work in the frontlines of healthcare about their leadership experiences and reflections. You have been identified as someone who would provide invaluable information about this topic. We anticipate that this interview will take about 30-45 minutes.

OVERVIEW OF INTERVIEW TOPICS

In this interview, we will ask you a series of open-ended questions to get your perspectives about several topics.

INTRODUCTION TO INTERVIEW

Before we begin the discussion, we need to take you through an informed consent process. In particular, let me make sure that you understand that:

1. Your participation is completely voluntary. If you do choose to talk with us, you may decide to end the conversation at any time.
2. We consider this discussion to be confidential. Your participation is confidential in the sense that your name will not be used in any reports or articles.
3. We would also like to record the session for the purposes of data collection for our research. The recording will not be used to identify you in any way.

Do you have any questions about our study or this interview process?

Interview Guide

- To start, can you please tell me about your current role?
- What challenges have you perceived health professionals like you experience in healthcare work environment?
- From your perspectives, why women HCWs leave the workforce early?
- From your perspectives, what workplace-based initiatives can support retention of workforce?
- What do you think can help address these challenges?
- Is there anything else you would like to share about your experience?

THANK YOU so much for your time and participation. Your participation was extremely helpful.

**Informed Consent**

Thank you for your participation in this study titled “Why they Persist in Health Workforce: A Mixed Method Study to Characterize and Examine the Experiences of Women”.

**Study Information and Consent Declaration**

**Background and Rationale**

Health systems around the world are facing major crisis related to health-care workforce (HCW). In 2021, Canada’s health-care labour market has seen an over 40 percent increase in job. This is affecting patient care in Canadian hospitals and long-term care facilities. Strategic human resources planning is required to ensure HCW retention and health system sustainability. This study aims to explore (1) why women HCWs leave the workforce early, (2) what workplace-based factors support their retention, and (3) the effects of interventions designed to retain women HCWs.

**Why were you selected to participate in this study?**

We are asking you to participate in this study because your role as a licensed clinician working in Ontario who can provide your perspective and expertise regarding your experiences in working in health care. Your active participation is highly valued, with potential benefits of improving work conditions for healthcare workforce.

What participation entails

We are cognizant of the impact Covid-19 has had on all who work in healthcare. We are mindful of the value of people’s time and energy and with that in mind endeavour to make participation in our study as efficient and beneficial as possible for our participants. Participation will involve:

- A virtual interview:
  - Approximately 45 minutes in length
  - Conducted at a time that is convenient for you
  - These interviews will be transcribed

You will receive a $60 gift certificate to recognize your contribution to this project.

**Benefits and Potential Harms**

We plan to utilize the results of our study to inform health professional organizations and healthcare employers to develop evidence informed programs to support health professionals. Sharing and recollecting your personal experience can trigger emotional reactions. You can choose to withdraw from the study at any point.

**Participation and Withdrawal**

Participation in this study is entirely voluntary. You can withdraw your participation at any time, which will not result in any negative consequences. All personal information collected will be protected in line with the University of Toronto Data Security Standards for Personally Identifiable and Other Confidential Data in Research.

**Privacy & Confidentiality**

Your interview responses will be kept strictly confidential. Unless otherwise required by law, the interview results will only be seen by the study team and the Research Ethics Board of University of Toronto (Toronto, Ontario, Canada) for the purpose of study monitoring.

Any identifiable information disclosed during the interview will be separated from your interview notes at the end of the study. The interview data will be stored securely. Recordings and notes will be accessible only to the principal investigator and study team and will be destroyed seven years after publication of this work. It is important to understand that despite these protections being in place, there continues to be the risk of unintentional release of information. However, any chance that this information will be accidentally released is very small.

**Ethics Approval**

This research has been reviewed and approved by the Health Sciences Research Ethics Board at the University of Toronto.

Who to Contact if You Have any Questions

If you have any questions about this study or your participation, you may contact Dr. Abi Sriharan ([abi.sriharan@utoronto.ca](mailto:abi.sriharan@utoronto.ca)). If you have any questions regarding your rights as a research participant, you may contact the Research Oversight and Compliance Office - Human Research Ethics Program at ethics.review@utoronto.ca or 416-946-3273, during regular business hours.

**Consent**

I have read the information above.

- I agree to participate in this study entitled “Why they Persist in Health Workforce: A Mixed Method Study to Characterize and Examine the Experiences of Women”
- I confirm that I am a licensed health professional.
- I will receive a $60 gift certificate at the completion of the interview.
- My personal information will remain anonymous.
- I understand and agree to give my consent to participate

**RECRUITMENT FORM**

Greetings,

We are conducting a study - Why they Persist in Health Workforce: A Mixed Method Study to Characterize and Examine the Experiences of Women," - to deepen our understanding of women's experiences in the healthcare workforce. More specifically, we will explore the following:

Why women HCWs leave the frontline clinical care early.

What workplace-based factors support their retention.

The effects of interventions designed to retain women HCWs.

We are asking you to participate in this study because of your role as a licensed clinician working in Ontario who can provide your perspective and expertise regarding your experiences in working in health care. Your active participation is highly valued, with the potential benefit of improving work conditions for the healthcare workforce.

What participation entails

We are mindful of the impact Covid-19 has had on all who work in healthcare. We are mindful of the value of people's time and energy and, with that in mind, endeavour to make participation in our study as efficient and beneficial as possible for our participants. Participation will involve:

o Approximately 45 minutes virtual (audio only)

o Conducted at a time that is convenient for you

o Interviews will be scheduled in January/February

o These interviews will be transcribed

After completing the interview, you will receive a $60 gift certificate for recognizing your contribution to this project.

Please complete this form if you are available to participate in this study. If you meet our eligibility criteria, a research team member will contact you shortly to schedule an interview.

Please do not hesitate to contact me if you have any questions.

Warm regards,

Dr. Abi Sriharan (womenhcp@gmail.com)

**DEMOGRAPHIC SURVEY**

**What is your current employment status?**

Full-time

Part-time

Seeking opportunities currently

Retired

Prefer not to say

**What is your annual employment income**

Less than $50,000

Between $50,000-$100,000

Between $100,000-$150,000

Between $150,000-$200,000

More than $200,000

Prefer not to say

**How many dependent children do you have?**

None

1-2

3-4

More than 4

Prefer not to say

**What is your age?**

Less than 30 years old

31-40 years old

41-50 years old

51-60 years old

61-70 years old

71+

Prefer not to say

**What is your cultural background? (Choose all that apply)**

African

European

East Asian

Southeast Asian

South Asian

First Nations or Indigenuous

Hispanic or Latinx

Middle Eastern

Prefer not to answer

**How long have you been work in healthcare**

Less than 1 year

1 to 5 years

6 to 10 years

11-15 years

More than 15 years

Prefer not to answer
